# SUS-BAR: a database of pig proteins with statistically validated structural and functional annotation

**DOI:** 10.1093/database/bat065

**Published:** 2013-09-21

**Authors:** Damiano Piovesan, Giuseppe Profiti, Pier Luigi Martelli, Piero Fariselli, Luca Fontanesi, Rita Casadio

**Affiliations:** ^1^Bologna Biocomputing Group, University of Bologna, via S. Giacomo 9/2, I-40126, Bologna, Italy, ^2^Department of Biological, Geological and Environmental Sciences (BIGEA), University of Bologna, via Selmi 3, I-40126, Bologna, Italy, ^3^Department of Computer Science and Engineering, University of Bologna, Mura A. Zamboni 7, I-40126, Bologna, Italy, ^4^Health Science and Technologies-ICIR, University of Bologna, Via Tolara di Sopra 41/E, I-40064, Ozzano dell’Emilia, Italy and ^5^Department of Agro-Food Science and Technology (DISTAL), University of Bologna, Viale Fanin 46, I-40127, Bologna, Italy

## Abstract

Given the relevance of the pig proteome in different studies, including human complex maladies, a statistical validation of the annotation is required for a better understanding of the role of specific genes and proteins in the complex networks underlying biological processes in the animal. Presently, approximately 80% of the pig proteome is still poorly annotated, and the existence of protein sequences is routinely inferred automatically by sequence alignment towards preexisting sequences. In this article, we introduce SUS-BAR, a database that derives information mainly from UniProt Knowledgebase and that includes 26 206 pig protein sequences. In SUS-BAR, 16 675 of the pig protein sequences are endowed with statistically validated functional and structural annotation. Our statistical validation is determined by adopting a cluster-centric annotation procedure that allows transfer of different types of annotation, including structure and function. Each sequence in the database can be associated with a set of statistically validated Gene Ontologies (GOs) of the three main sub-ontologies (Molecular Function, Biological Process and Cellular Component), with Pfam functional domains, and when possible, with a cluster Hidden Markov Model that allows modelling the 3D structure of the protein. A database search allows some statistics demonstrating the enrichment in both GO and Pfam annotations of the pig proteins as compared with UniProt Knowledgebase annotation. Searching in SUS-BAR allows retrieval of the pig protein annotation for further analysis. The search is also possible on the basis of specific GO terms and this allows retrieval of all the pig sequences participating into a given biological process, after annotation with our system. Alternatively, the search is possible on the basis of structural information, allowing retrieval of all the pig sequences with the same structural characteristics.

**Database URL:** http://bar.biocomp.unibo.it/pig/

## Introduction

In recent years, significant progress has been made in pig genomics due to the integration of modern sequencing techniques and computational biology methods (1–4). The collaborative effort of researchers in the swine genomics community is ongoing for a complete annotation of the pig genome (http://piggenome.org) (4). A major problem is how to understand whether pig proteins that are already available are informative enough to be useful in the annotation process (5, 6). Even though a given protein is translated and expressed, the annotation of the protein’s structure and function may not be straightforward. A similarity search where proteins with no annotation are analysed by their similarity to proteins with known annotation can be routinely performed to attribute structural and functional annotation to the unknown protein (5–8). The notion behind this procedure is that protein structure is more conserved than sequence through evolution, a very general concept that helps in modelling proteins with similar sequences as long as their sequence identity (SI) is ≥30% over the alignment length (9, 10). This observation is at the basis of building by comparison, one of the most successful methods for computing the 3D structure of a protein sequence when a template is found with a sequence similarity search against the Protein Data Bank (PDB) (11). Functional properties are, however, more difficult to be transferred. According to current literature, they are generally inferred on the basis of structural conservation and it is commonly accepted that proteins sharing approximately 40–60% of sequence identity are also likely to share similar function (12, 13). When SI is < 30%, proteins are categorized to be distantly related to their homologous counterparts when they perform the same function in different organisms. In this case, they can possibly share the same structure although sharing very little SI (14, 15). Distantly related homologues can be recognized by methods that model structural and functional domains, such as Pfam (http://pfam.sanger.ac.uk) (16). When a protein sequence significantly matches a specific Pfam model, the Pfam model-associated function is also transferred to the target protein. Functions are routinely described with specific terms following the Gene Ontology (GO), comprising three main functional routes: Molecular Function, Biological Process and Cellular Component (17). UniProt Knowledgebase (UniProtKB), one of the largest resources of protein sequences, automatically curates annotated protein records (http://www.uniprot.org/help/biocuration) (18). Here annotation integrates previous knowledge on protein structure and function from various sources, when available, mainly based on profile and Hidden Markov Model (HMM) methodologies (UniProtKB/TrEMBL; http://www.ebi.ac.uk/GOA). Therefore, protein sequences may be endowed with a wealth of featured data that include extensive cross-reference to other specialized databases (including PDB and Pfam), experimentally detected or predicted functional and structural patterns and assigned functional GO terms. Protein records are manually curated in the SwissProt section of UniProtKB (UniProtKB/SwissProt). Most of the sequence entries are proteins that have only been recognized on the basis of sequence similarity or predicted without any experimental evidence of their existence (http://www.ebi.ac.uk/uniprot/TrEMBLstats/). This is also the case for majority of the currently available pig proteins, whose annotation relies mainly on automatic procedures.

In this article, we describe SUS-BAR, a database that collects all the available pig protein sequences and provides statistical validation and enrichment to the GO functional terms and Pfam domains that are present in the UniProtKB files. Statistical validation is obtained with a cluster-centric method that has been described before (19–21) and that also allows structural modelling of the protein sequence, when possible. Our method has been recently and successfully benchmarked in one experiment of Automated Function Prediction featuring a Critical Assessment of Function Annotations [AFP/CAFA 2011, (21, 22)]. Adopting our procedure, we provide statistically validated GO and Pfam terms to some 63% of the all pig protein set. Furthermore, we provide structural templates to 27% of pig protein sequences. We newly annotate 1283 pig protein sequences not annotated before with GO terms and/or Pfam domains in UniProtKB. In SUS-BAR, pig protein sequences can be associated to statistically validated GO terms of the three main branches, to statistically validated Pfam domains and, when possible, also to structural templates to build their 3D structural model. The database is freely available and downloadable at http://bar.biocomp.unibo.it/pig. Alternative ways of search for retrieving information over the pig proteome, including also the possibility to search pig proteins in relation to other related and unrelated organisms, are available.

## Databases and methods

### Database

The dataset includes 26 134 different pig protein sequences downloaded from UniProtKB (2013_01 Release) and selected from the complete proteome set. Another 72 unique sequences were collected from the Ensembl 70 genebuild based on *Sus scrofa* 10.2 pig genome assembly (http://www.ensembl.org). The SUS-BAR database collects 26 206 sequences.

### SUS-BAR

SUS-BAR is the Bologna Annotation Resource for *Sus scrofa* that we implement. It is based on our annotation system BAR+ (available at http://bar.biocomp.unibo.it/bar2.0). BAR+ allows transfer of statistically validated annotation, and it has been described before (19–22). Briefly, the method is based on the concept that sequences can inherit the same function/s and structure from their well-annotated counterparts, provided that they fall into the same cluster, endowed with statistically validated GO terms and Pfam domains. For generating BAR+ clusters, we analysed >13 million protein sequences from 988 genomes and UniProtKB release 2010_05. The BAR+ cluster building pipeline starts with an all-against-all sequence comparison with BLAST in a distributed unified computing resource (GRID environment, http://www.cnaf.infn.it/it/users/grid) (20). In the present version, we complement our previous BAR+ with additional 30 000 sequences downloaded from UniProtKB/SwissProt release 2012_01, including human protein variants. The clustering procedure constrains SI to be ≥40% on at least 90% of the global alignment length (Coverage). The alignment results are regarded as an undirected graph where nodes are proteins and links are allowed only among chains that are 40% identical over at least 90% of the alignment length. A cluster comprises all the connected protein nodes (19). In the present implementation, we count 1 254 869 clusters containing 82% of all the protein sequences of BAR+. When a cluster incorporates a UniProtKB entry, it inherits its annotations [GO and Pfam terms, PDB structures and Structural Classification of Proteins (SCOP)]. Within a cluster, GO and Pfam terms are then statistically validated as previously described (19). A *P*-value is computed with a Bonferroni-corrected procedure after setting a threshold value for significance with a bootstrapping technique (19). Statistically validated terms are those endowed with *P* < 0.01 (19, 20). In this version, some 383 679 clusters contain statistically validated terms, and ∼12 million protein sequences fall into these statistically validated clusters. Clusters can contain distantly related proteins that can, therefore, be annotated with high confidence and eventually can also inherit a structural template, if present. Structural alignments within each cluster-containing templates are provided by a cluster HMM and are available for downloading (20). Depending on the annotation types of the sequences within the cluster, all new targets that fall into a cluster can inherit all the cluster statistically validated annotations by transfer.

In SUS-BAR, GO terms are directly retrieved from UniProtKB. In UniProtKB, most of the GO terms are inferred from electronic annotation. However, and more importantly, GO terms with some experimental validation are also present [labels: EXP, IDA, IPI, IMP, IGI, IEP (see http://www.geneontology.org/GO.evidence.shtml)]. The cluster-centric procedure of SUS-BAR emphasizes their presence after their statistical validation. Most of these terms derive from sequences present in the SwissProt section of UniProtKB. Therefore, each cluster is highlighted with a green dot and a red dot when it contains statistically validated and SwissProt-derived annotation. Furthermore, a yellow star in the cluster indicates when GO terms with some experimental and statistical validation are present. Within the cluster, the yellow star is also associated to each specific statistically validated GO term labelled with an experimental evidence code.

After this procedure, we re-collected all the pig proteome, and for each sequence, we collected the annotation as derived from statistically validated clusters, non-validated clusters and singletons. This information is contained in SUS-BAR, it is downloadable and it can also be retrieved upon search over the database.

## Results and Discussion

### The current annotation of the pig proteome

In Table 1, the total number of pig proteins, as retrieved from the present releases of the databases, is sorted out based on the number of sequences endowed with unique GO of the three main roots (Molecular Function, Biological Process and Cellular Component) with all the GO terms (All-GO), Pfam domains (Pfam), both Pfam and All-GO terms and a structure in the PDB. Sequences are also listed depending on the UniProtKB branch from where they were retrieved (SwissProt, manually annotated and reviewed, and TrEMBL, automatically annotated). Pfam terms are derived from UniProtKB that links directly to the Pfam database ((16), http://pfam.sanger.ac.uk). Summing up, only 107 pig protein sequences have associated PDB structure with atomic resolution, 68% of the sequences are endowed with 4225 Pfam domains (Pfam), 68% of the sequences are endowed with 9809 GO terms of the three main branches and 79% of the sequences are annotated with Pfam domains and GO terms (“Pfam and All-GO” column in Table 1). With the exception of the proteins listed in SwissProt, most of the annotation largely derives from feature transfer mainly based on profile and HMM methodologies (UniProtKB/TrEMBL; http://www.ebi.ac.uk/GOA). In the UniProtKB files, ∼92% of the whole set of pig protein sequences either have protein homologues based on some extent of similarity with previously annotated proteins in the database or are predicted.

**Table 1.** Annotation of the PIG proteome in UniProtKB and Ensembl

Dataset	MFO	BPO	CCO	All-GO	Pfam^a^	Pfam and All-GO	PDB^b^
SwissProt (1482)^c^							
Sequences	1065	1111	1331	1395	1298	1402	104
Terms	811	2117	340	3268	986	4254	–
TrEMBL (24 652)^c^							
Sequences	12 451	11 537	12 349	16 357	16 559	19 284	1
Terms	1936	6443	913	9292	4138	13 430	–
Ensembl (72)^c^							
Sequences	47	22	25	49	45	52	2
Terms	58	107	35	200	43	243	–
Total (26 206)^c^							
Sequences	13 563	12 670	13 705	17 801	17 902	20 738	107
Terms	2190	6678	941	9809	4225	14 034	–

UniProtKB release: 2013_01; Ensembl release: Ensembl 70 genebuild based on *Sus scrofa* 10.2 pig genome assembly.

ALL-GO: number of sequences with MF *OR* BP *OR* CC.

^a^Pfam domains. Union of ALL-GO and Pfam.

^b^PDB: protein pig sequences with a correspondent PDB structure.

^c^Number of PIG protein sequences. Numbering considers only unique GO terms and Pfam domains.

MFO, molecular function ontology; BPO, biological process ontology; CCO, cellular component ontology.

### SUS-BAR validates and enriches the pig proteome annotation

With our method, all the pig protein sequences are aligned towards the BAR+ database and they may enter into a cluster containing statistically validated information (*P* < 0.01) for a specific GO term or Pfam domain. This is the case for 25 989 pig protein sequences while 217 remain singletons and carry along the UniProtKB or Ensembl annotation (when present). Sixty-four percent of the cluster-retained sequences align towards clusters endowed with statistically validated annotation, and they inherit all the cluster statistically validated GO terms and/or Pfam domains. When a cluster template/s is/are available, the sequence may benefit from a pre-computed cluster HMM that allows its structural modelling. The results are shown in Table 2. It appears that after BAR+ alignment, the number of annotation terms inherited by the sequences in a validated manner surpasses the same features described in Table 1. Summing up, some 16 675 pig sequences by falling into 9552 SUS-BAR clusters inherit 21 901 validated Pfam and GO terms. The large increase in the number of statistically validated GO terms inherited by the pig sequences with our method is particularly evident when the All-GO columns in Tables 1 and 2 are compared.

**Table 2.** Statistically validated annotation of the pig proteome in SUS-BAR

Dataset	MFO	MFO^a^	BPO	BPO^a^	CCO	CCO^a^	All-GO	All-GO^a^	Pfam	Pfam and All-GO	^b^PDB
Cluster (25 989)^c^^,d^											
Sequences	12 755	9147	13 611	11 323	13 749	11 480	15 500	12 918	15 488	16 675	7284
Clusters	6491	3835	7064	5344	7155	5496	8597	6523	8598	9552	3421
Terms	3902	3215	12 520	12 020	1517	1370	17 939	16 605	3962	21 901	–
Singleton (217)^c^^,d^											
Sequences	121	8	131	9	167	9	179	9	133	181	0
Terms	132	18	222	19	79	9	433	46	118	551	–
Total (26 206)^d^											
Sequences	12 876	9155	13 742	11 332	13 916	11 489	15 679	12 927	15 621	16 856	7284
Terms	3904	3218	12 521	12 020	1517	1370	17 942	16 608	3968	21 910	–

^a^Terms that are statistically validated and have an experimental evidence code with the corresponding number of sequences that inherit them in a given number of clusters.

^b^Pig protein sequences in clusters that inherit a structure.

^c^Numbering considers only unique GO terms and Pfam domains.

^d^Clusters are generated as described in the SUS-BAR section. Singletons are pig sequences that do not belong to clusters and carry along only their original UniProtKB or Ensembl annotation.

In Table 2, we address how much of the transferred annotation is also endowed with an experimental evidence code. Evidently, 75% of the clusters containing statistically validated terms also contain GO terms that are endowed with an experimental evidence code. SUS-BAR clusters (18 738) are mostly (49%) seeded on SwissProt sequences that carry along most of the experimental information (PDB, Pfam, GO annotations, including the experimental ones). In SUS-BAR, 75% of the clusters contain statistically validated GO annotations endowed with an experimental evidence code (All-GO^a^/All-GO, Table 2). Approximately 83% of the pig sequences present in statistically validated clusters inherit statistically validated GO annotations that are also characterized by an experimental evidence code. Moreover, and more importantly, GO annotations are corroborated by structural and functional annotations (Pfam domains, PDB templates). In our system, a pig sequence can, therefore, be annotated also by checking whether the GO annotation is consistent with Pfam domains that the sequence may inherit and/or by modelling the sequence on a given template. A possible way of computing the increase in GO terms attributed by BAR+ as compared with UniProtKB is by considering direct annotations as well as their respective ancestor terms: 9156 of the 17 939 statistically validated GO terms (All-GO column in Table 2) were already present (although without statistical validation) in UniProtKB. By counting both, direct annotations and their respective ancestor terms, we obtain 150 992 and 345 843 GO terms starting from those of UniProt and ours, respectively. This corresponds to a 2.3-fold increase in GO terms obtained when BAR+ is adopted, including singletons that retain their original UniProtKB and Ensembl annotation.

Furthermore, considering our data at the different protein sequence levels (downloadable at http://bar.biocomp.unibo.it/pig/download.htm), with BAR+, 10 559 pig proteins acquire 16 076 statistically validated new GO terms that were not present in UniProtKB or in their ancestors terms; 5461 pig proteins acquire 7242 GO terms that are more specific than those reported in the corresponding UniProtKB files (GO terms that are descendents of the UniProtKB GO terms). The number of clusters with at least one SwissProt entry in SUS-BAR is 9219 over a total of 18 738.

With our method it is also possible to model distantly related targets that fall into a cluster by means of a cluster HMM. By aligning towards 3421 clusters enriched with cluster HMMs (Table 2), 7284 pig protein sequences also inherit template structures. Thirty-five percent of these pig targets share an SI < 30% with the template structure/s of the cluster and their modelling would be impossible based only on sequence similarity search. Concomitantly, each sequence also inherits statistically validated Pfam domain and GOs, allowing mapping of the functional annotation directly on the protein model.

In Table 3, the effect of our annotation procedure is shown for sequences without any annotation in UniProtKB and Ensembl. A small fraction of this subset (1283 protein pig sequences) is aligned towards SUS-BAR clusters characterized by statistically validated annotation and is therefore annotated. Sixty-eight percent of the set inherits GO annotations that are endowed with an experimental evidence code. Five hundred sixty-seven sequences also inherit structural templates. The remaining sequences fall into clusters that are not validated. In SUS-BAR, the UniProtKB and Ensembl links are also reported for singletons.

**Table 3.** SUS- BAR annotation of pig protein sequences not annotated with GO terms and/or Pfam domains in UniProtKB and Ensembl

Dataset	MFO	MF^a^	BPO	BP^a^	CCO	CC^a^	All-GO	All-GO^a^	Pfam	Pfam and All-GO	PDB^b^
UniProtKB (5448)^c^											
Sequences	795	539	908	716	952	732	1118	859	903	1209	558
Clusters	541	345	645	492	670	493	806	594	658	892	365
Terms	1306	1044	6684	6303	798	685	8788	8032	581	9369	–
Ensembl (171)^c^											
Sequences	10	6	21	10	29	18	34	20	62	74	9
Clusters	10	6	20	9	28	18	32	19	50	62	8
Terms	37	20	306	264	117	89	460	373	45	505	–
Total (5619)^c^											
Sequences	805	545	929	726	981	750	1152	879	965	1283	567
Clusters	547	349	660	500	692	507	832	609	706	948	372
Terms	1311	1046	6694	6313	811	697	8816	8056	621	9437	–

^a^Terms that are statistically validated and have an experimental evidence code with the corresponding number of sequences that inherit them in a given number of clusters.

^b^Inherited with cluster HMMs.

^c^Number of pig protein sequences in the two databases.

### How to search SUS-BAR

**Figure 1.**
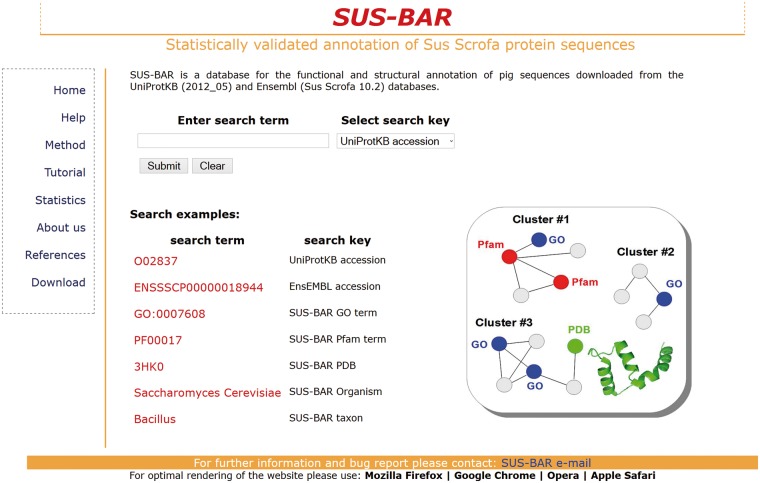
The SUS-BAR interface. Query requires both a search term and the selection of the corresponding search key (http://bar.biocomp.unibo.it/pig).

The database underlying the SUS-BAR Web site allows two types of searches depending on the information available to the user (Figure 1). When for a given pig protein, the UniProtKB or Ensembl accession is known, the Web site returns the protein and the corresponding cluster identification code (when the sequence is not a singleton). Then the user can explore all the annotation of the corresponding cluster by clicking on the cluster field (see Tutorial on the Web site, http://bar.biocomp.unibo.it/pig/tutorial.htm).

SUS-BAR also makes it possible to perform a search based on keywords. This allows the retrieval of the pig protein sequences from clusters containing the keyword.

The search allows directly pasting any GO term of interest. The site will return the list of all pig proteins that fall into clusters where the specific GO term is statistically validated. It is also possible to search for pig proteins that fall into clusters where other protein sequences from a specific organism or taxon are co-located. For instance, if the user searches for ‘Cetartiodactyla’ under the ‘SUS-BAR taxonomy’ field, the Web site returns all the pig protein sequences that fall into clusters where at least one member of the population contains the string ‘Cetartiodactyla’ in its taxonomy (see Tutorial on the Web site, http://bar.biocomp.unibo.it/pig/tutorial.htm). GO annotations endowed with an experimental evidence code are labelled with a yellow star. Clusters statistically validated and containing SwissProt sequences are labelled with a green and a red dot, respectively.

### Searching by organism

The rationale behind our database offers the unique opportunity to retrieve per organism all the clusters where pig protein sequences are grouped with those from other organisms. In Table 4, the search by *Homo sapiens*, *Mus musculus* and *Bos taurus* retrieves all the clusters where sequences of the three organisms share some annotation with those of the pig, including, when available, a structural template. Interestingly, a large fraction of the pig protein sequences inherit statistically validated annotation from the clusters, albeit low sequence identity (SI < 30%), with sequences carrying information into the cluster.

**Table 4.** Pig sequences in clusters with other organisms

Organism	Number of clusters	Number of Pig sequences	Number of Pig sequences (SI < 30%)	Number of clusters with PDB	Number of Pig sequences inheriting PDBs
*Homo sapiens*	9409	16 480	1288	2732	6013
*Mus musculus*	8804	15 771	579	823	2461
*Bos taurus*	8413	15 340	148	213	758

SI, sequence identity.

Another search option allows retrieval of some 100 pig sequences that can be modelled with 67 cluster HMMs whose structural templates are derived from distantly related prokaryotic sequences (the list of these templates and the corresponding pig protein is included as Supplementary Material).

### Searching by GO terms

**Figure 2.**
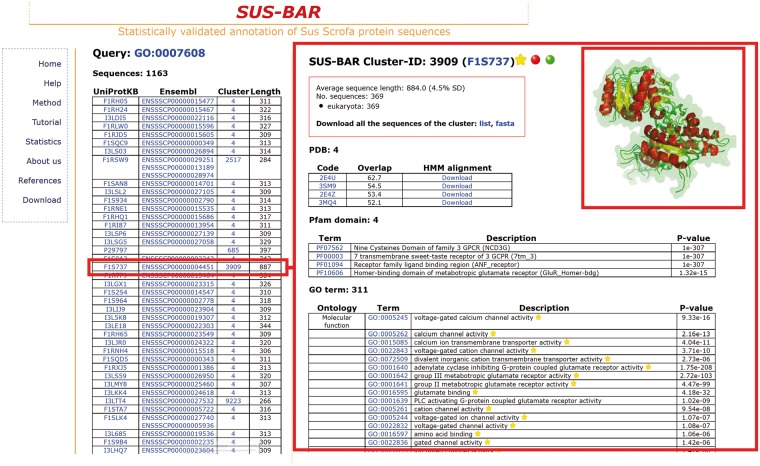
SUS–BAR sequences endowed with statistically validated GO terms corresponding to the sensory perception of smell. The left panel lists all the pig sequences that are retrieved when the database search is done with GO:0007608. In all, 1163 pig sequences in SUS–BAR inherit the same statistically validated GO term by entering clusters where the term is statistically validated by computing its Bonferroni-corrected *P*-value (19). Characteristics of the cluster (#3909) where one of these 1163 pig protein sequences (F1S737, red box in the left) is located are shown in the right panel. Structural alignment of the four PDBs contained in the clusters is done with Mustang (25) and visualized with PyMol (http://www.pymol.org). The inset is manually computed for the figure and is not present in the corresponding page of the Web site. Each pig protein sequence in the cluster can be, however, modelled in house after downloading of the corresponding alignment with the templates in the cluster. This is provided with the cluster-specific HMM. See text for further details. Yellow stars indicate that the GO term is statistically validated and endowed with an experimental evidence code. The red dot indicates that the cluster contains SwissProt annotated protein/s.

One important problem in pig breeds is the sensory perception of smell, a biological process that comprises all the events required for an animal to receive an olfactory stimulus, convert it to a molecular signal and recognize and characterize the signal (23, 24). The corresponding GO term is GO:0007608, and it only characterizes the pig odorant-binding protein in UniProtKB/SwissProt (code P81245, OBP_PIG with a corresponding PDB structure covering the whole protein sequence) and 11 other sequences in the current version of Ensembl. We find that another 1163 pig protein sequences in 17 clusters inherit the same statistically validated GO term (Figure 2, left) and can, therefore, be included into the same biological process. In the right panel of Figure 2, we show details of one (F1S737) of the retrieved pig protein sequences located in cluster #3909. The cluster contains 369 sequences from 53 eukaryotic organisms and 4 PDB structures of neurotransmitter metabotropic glutamate receptors from two different eukaryotic species [3SM9 and 3MQ4 from *Homo sapiens* (Q14831, Q14832) and 2E4U and 2E4Z from *Rattus norvegicus* (P35400, P31422)]. Structures overlap with a root-mean-square deviation of 0.144 nm and share the same fold. They are also used to generate the corresponding profile-based cluster HMM for modelling all sequences that belong to the cluster, including the pig one, from where they acquire annotation (the UniProtKB file F1S737 annotated the same protein as uncharacterized). The cluster also contains four statistically validated Pfam domains and other 311 statistically validated GO terms of the three different branches. All the pig protein sequences that are retrieved and are endowed with a statistically validated GO term that is associated to a specific biological process may belong to the network characterizing the process in the animal. The list of the pig proteins that, according to the present version of SUS-BAR, are involved in the pig sensory perception of smell is shown in the left panel of Figure 2 and can be downloaded for further experimental validation.

## Conclusion

We present SUS-BAR, a database where approximately 64% of the currently available pig proteome is endowed with statistically validated GO terms and Pfam domains. In the genomic era, most of the protein sequences are derived from the direct translation of the coding sequences after genome annotation. Therefore, functional and structural annotation of the proteins is urgent to corroborate their existence at the cell level. When annotation is done electronically, a robust validation process can help in the inheritance of Pfam and GO terms by transfer of annotation. Here, we make use of a cluster-centric system of annotation and generate SUS-BAR where enrichment in GO terms and Pfam is ensured after a robust statistical validation of the annotation. By this a large fraction of the pig proteome is endowed with structural and functional features that will help in designing future validation experiments. Furthermore, we can endow approximately 27% of the whole pig proteome with structural models. At least 35% of the proteins that inherit a structural model in SUS-BAR share <30% sequence similarity with the template/s, indicating that with our procedure also distantly related homologues can be safely annotated. SUS-BAR will be updated when new releases of the pig genome in the Ensembl and UniProtKB/SwissProt databases will become available. SUS-BAR will also be updated following the major updates of BAR+. All the pig protein sequences with statistically validated GO (including those with an experimental evidence code) annotations and associated Pfam domains are freely downloadable from the SUS-BAR Web site.

## Supplementary data

Supplementary data are available at *Database* Online.

## Funding

This work was supported by the Italian Ministry for University and Research: MIUR (PRIN 2009 project 009WXT45Y, PON project PON01_02249); European Union RTD Framework Programme (COST BMBS Action TD1101); 2007-2013 Regional Operational Programme of the European Regional Development Fund (ERDF) and the Emilia-Romagna region. D.P. was a recipient of a PHD fellowship from the Ministry of the Italian University and Research.

*Conflict of interest*. None declared.

## Supplementary Material

Supplementary Data
