# Supplementary material for: SUS-BAR: a database of pig proteins with statistically validated structural and functional annotation
Source: Database (Oxford). 2013 Sep 21;2013:bat065. doi: 10.1093/database/bat065 (PMC3781388; doi:10.1093/database/bat065)
Supplement: Supplementary Data [file supp_2013_bat065_index.html]

Supplementary Data 

# SUS-BAR: a database of pig proteins with statistically validated structural and functional annotation

## Supplementary Data

files

**Files in this Data Supplement:**

- Supplementary Data - xls file
